# MicroRNA Regulation of Adipose Derived Stem Cells in Aging Rats

**DOI:** 10.1371/journal.pone.0059238

**Published:** 2013-03-14

**Authors:** Jia Fei, Holly Tamski, Carla Cook, Nalini Santanam

**Affiliations:** Department of Pharmacology, Physiology and Toxicology, Joan C. Edwards School of Medicine, Marshall University, Huntington, West Virginia, United States of America; University of Louisville, United States of America

## Abstract

**Background:**

Perturbations in abdominal fat secreted adipokines play a key role in metabolic syndrome. This process is further altered during the aging process, probably due to alterations in the preadipocytes (aka. stromal vascular fraction cells-SVF cells or adipose derived stem cells-ASCs) composition and/or function. Since microRNAs regulate genes involved both in development and aging processes, we hypothesized that the impaired adipose function with aging is due to altered microRNA regulation of adipogenic pathways in SVF cells.

**Methodology and Principal Findings:**

Alterations in mRNA and proteins associated with adipogenic differentiation (ERK5 and PPARg) but not osteogenic (RUNX2) pathways were observed in SVF cells isolated from visceral adipose tissue with aging (6 to 30 mo) in female Fischer 344 x Brown Norway Hybrid (FBN) rats. The impaired differentiation capacity with aging correlated with altered levels of miRNAs involved in adipocyte differentiation (miRNA-143) and osteogenic pathways (miRNA-204). Gain and loss of function studies using premir or antagomir-143 validated the age associated adipocyte dysfunction.

**Conclusions and Significance:**

Our studies for the first time indicate a role for miRNA mediated regulation of SVF cells with aging. This discovery is important in the light of the findings that dysfunctional adipose derived stem cells contribute to age related chronic diseases.

## Introduction

The physiological functions of adipose tissue are not restricted to being a lipid storage organ but also to serve as an endocrine organ that secretes cytokines and hormones involved in lipid and glucose metabolism [1]. Adipose tissue is primarily composed of ‘preadipocytes’, and other cellular fractions including immune cells. Histologically, ‘preadipocytes’ derived from stromal vascular fraction cells (SVF cells) are also known as ‘adipose derived stem cells (ASC)’ or ‘adipose derived mesenchymal stem cells’ [2]–[4]. These are defined as the cellular population with multilineage potential with neurogenic, adipogenic, chondrogenic and osteogenic differentiation capabilities [5]–[7]. Though these cells represent only a very small population in localized tiny niches in the adipose tissue, due to their increased capacity for self-renewal and multilineage differentiation, they are the major source of mature adipocytes [8].

The preadipocyte fraction of the adipose tissue modulates the endocrine function of the adipose tissue [9]. When the adipose tissue mass changes, either due to increase in weight gain or other physiological alterations, there is an increased secretion of pro-inflammatory adipokines from visceral fat. This increase in secretions and subsequent alterations in lipid homeostasis and insulin resistance [10], [11] can lead to obesity and higher risk for cardiovascular diseases [12]–[14]. Physiological aging also dramatically alters adipose tissue mass, distribution and function [15] [2] [16]. However, in spite of these changes *in vivo*, the preadipocytes isolated from various age groups of animals or fat-depots (subcutaneous versus visceral or epicardial) retain their phenotypic characteristics even after excision and growth in *ex-vivo* culture [17] [3]. We recently showed significant changes in adipose gene expression in a sex and fat-depot specific manner, with increase in age [18]. This age associated alteration in adipose function might be attributed to changes in ASC composition and function.

The differentiation capacity of ASCs is transcriptionally regulated by PPARγ (peroxisome proliferator activated receptor g) and Runx2 (Runt-related transcription factor 2), the two reciprocal switches for the adipogenic and osteogenic pathways [19]. PPARγ is the major player in adipocyte differentiation [20]. Runx2, on the other hand, switches mesenchymal stem cell differentiation to bone cell lineage prior to the expression of osteoblastic phenotype [21]. Recently, microRNAs (miRNA), which are small nucleotide (17–20 nt) non-coding RNAs that play a regulatory role in mRNA transcription and translation [22], have been identified to regulate both the adipogenic and osteogenic pathways [7], [23]–[25]. miR-143 through its actions on its target genes in the ERK5-PPARγ pathway, promotes adipogenesis and obesity [26]. Likewise, miR-204 inhibits osteogenic differentiation of mesenchymal stem cells through direct suppression of Runx2 [27]. Aging or senescence decreases adipogenic but maintains osteogenic capacity of preadipocytes [28]. However, the mechanism by which aging or senescence modulates these two pathways and affects adipose tissue function is still unclear. We hypothesize that impairment of the adipogenic miRNAs with aging contributes to the imbalance between the adipogenic/osteogenic differentiation capacities resulting in altered preadipocyte function. The role of miR-143 and miR-204 and its target genes on the altered preadipocyte function in young (6 months old) and old (30 months old) Fischer 344 x Brown Norway Hybrid (FBN) rats were studied.

### Ethics statement

This study was carried out in strict accordance with the recommendations in the *Guide for the Care and Use of Laboratory Animals* of the National Institutes of Health. The protocol was approved by the Committee on the Ethics of Animal Experiments of Marshall University (Assurance # A3578-01). All animals were euthanized before tissues were excised and all efforts were made to minimize suffering.

## Materials and Methods

### Isolation and characterization of stromal vascular fraction (SVF) that contains ASCs

The visceral abdominal fat (intra-gonadal fat) was excised from 16 young and old female (n  = 8/group) (6- and 30- months (mo) old) Fischer 344 x Brown Norway hybrid rats (FBN) obtained from National Institutes on Aging. Animal ages were chosen on the basis of previous data demonstrating that these ages correspond roughly to women in their third (6 month rats) or eighth (30 mo rats) decade of life [29]. Animals were fed with a standard laboratory diet (rat chow) and water ad libitum until the time of sacrifice. After 2 weeks of acclimatization, the rats were sacrificed and visceral abdominal fat were excised. This study was carried out in strict accordance with the recommendations in the *Guide for the Care and Use of Laboratory Animals* of the National Institutes of Health. The protocol was approved by the Committee on the Ethics of Animal Experiments of Marshall University (Assurance # A3578-01). All animals were euthanized before tissues were excised and all efforts were made to minimize suffering.

Approximately 100 mg of fresh abdominal fat were minced to smaller pieces and incubated with Type II collagenase for 30 minutes (mins). The digested tissue was centrifuged at 2000 rpm for 5 mins to separate the floating population of mature adipocytes from the pelleted stromal vascular fraction (SVF) (abundant in preadipocytes). The supernatant obtained after the centrifugation step was aspirated and the pellet was suspended in a maximum of 3 ml stromal medium (DMEM+10% heat-inactivated fetal bovine serum (FBS) and 1% penicillin/streptomycin (P/S)). The cell suspension was filtered through a 70 µm mesh strainer [30]. The cells in the SVF were cultured and identified as preadipocytes using immunostaining and flow cytometry analysis.

Preadipocytes or adipose-derived stem cells (ASC) derived from stromal vascular fraction (SVF) cells, express a cluster of immune markers with relatively low specificity. The presence of these markers are species specific since it has been shown, unlike human adipose derived stem cells, the murine adipose derived stem cells express cell surface markers, CD90 and CD44 but very little CD45 and CD34 [25]. In this study, we used CD90, CD44 and CD34 as cell surface markers for identification of preadipocytes. Briefly, the isolated SVF cells were cultured in 35 mm dishes for over a week. At the end of a week, majority of the attached cells are adipose derived stem cells [30]–[33]. The cultured cells were analyzed for stem cell markers by immune-fluorescence staining using standardized protocols with antibodies specific to cell surface markers, CD90 (sc-53116) and CD34 (sc-7324) (Santa Cruz Biotechnology, CA). The immunostaining was detected using Leica Fluorescence microscope.

For flow cytometry detection of stem cell markers, preadipocytes from 6 mo and 30 mo old FBN rats at passage 5 were collected in fluorescence-activated cell sorting (FACS) buffer and incubated in 10% fetal calf serum for 20 minutes at 4°C. The cells were washed in FACS buffer prior to the antibody incubations. In order to detect the expression of CD90, CD34, and CD44 cell surface markers, samples of 300,000 cells were prepared at an antibody concentration of 1 µg/million cells for PE-conjugated mouse Anti-rat CD90/mouse CD90.1 (554898, BD Pharmingen™), PCP-Cy5-conjugated CD34 (sc-7324 PCPC5, Santa Cruz Biotechnology, Inc), and FITC-conjugated mouse anti-rat CD44H (550994, BD Pharmingen™) monoclonal antibodies, against a peroxidase-conjugated anti-mouse IgG (Sigma) secondary antibody. The cells were suspended in 500 µL of FACS buffer and analyzed using a BD FACS Aria Flow Cytometer. Data collected was analyzed using FlowJo 10.0 software.

The preadipocyte specific marker, preadipocyte factor-1 (Pref-1) was detected using the mRNA isolated from SVF cells and whole adipose tissue by real time-PCR (RT-PCR) using primers specific for Pref-1 (U25680): 5′-gaaccatggcagtgtgtctg-3′, 3′-agggagaaccattgatcacg-5′.

### Genomic DNA isolation and Quantitative Real-Time Polymerase Chain Reaction (RT-qPCR) for telomere length

Progressive shortening of telomere length with aging is considered a phenotypic marker of normal physiological aging [34]. Telomere length was measured in the genomic DNA isolated and purified from 5×10^5^ preadipocytes after DNA extraction (Qiagen, Valencia, CA). The telomere (T) length and single copy gene (S) was determined by RT-qPCR. The duplicate measurements of the T/S ratio in the same DNA sample gave a relative difference in telomere length comparison between different groups. Briefly, 35 ng of DNA was mixed with either the T or S primer and the PCR reagent followed by RT-qPCR on MyiQ Bio-Rad Real Time PCR system (Bio-Rad, Hercules, CA). The primer sequences used were: (5′→3′): T1, ggtttttgagggtgagggtg agggtgagggtgagggt; T2, tcccgactatccctatccctatccctatccctatcccta; 36B4u, cagcaagtggg aaggtgtaatcc; 36B4d, cccattctatcatcaacgggtacaa [35]. The data obtained are expressed as relative expression of fold change ±SEM (Standard Error of the Mean).

### Adipogenic or osteogenic differentiation of isolated preadipocytes

Differentiation capacity of the isolated preadipocytes from young and old rats was determined by culturing the cells in two specific differentiation media: (i) *Adipogenic differentiation*: As soon as preadipocytes (5×10^5^) in culture reached 100% confluence, the cells were transferred to an adipogenic induction media consisting of: 0.5 mM isobutylmethylxanthine, 0.5 µM insulin and 0.5 µM dexamethasone. The media was changed every 2–3 days for a total of 12 days, until adipocytes were visible. The differentiated adipocytes were recognized by the appearance of red-stained lipid droplets (a marker for differentiated adipocytes) in the presence of 0.5% Fat-Red-O staining. (ii) *Osteogenic differentiation*: 80% confluent preadipocytes were transferred to an osteogenic induction media consisting of 1 nM dexamethasone, 2 mM β-glycerolphosphate and 50 µM ascorbate-2-phosphate for 14 days with a media change every 3 days. Osteogenic differentiation was confirmed by the appearance of mineralization as assessed by the positive appearance of an orange-red color after staining with 40 mM Alizarin Red (pH 4.1).

The extent of adipogenic or osteogenic differentiation of the preadipocytes was quantified using IMAGEJ PC-based software (National Institute of Health, NIH Version v1.32j).

### RNA isolation and RT-qPCR for miRNA and mRNA detection

Total RNA was isolated by homogenization of the preadipocytes (5×10^5^) or 100 mg of whole adipose tissue on ice with ml Tri-reagent as directed by the manufacturer (Sigma, St. Louis, MO). RNA concentration and purity were analyzed using Nanodrop model 1000 (Nanodrop, Wilmington, DE) while RNA integrity was confirmed using 1.2% agarose gel electrophoresis followed by RIN analysis on the Agilent Bioanalyzer.

#### mRNA detection

Purified RNA (1 µg) was utilized for the synthesis of complementary DNA (cDNA) using iScript cDNA synthesis kit (Bio-Rad, Hercules, CA). Real-time PCR was carried out in 25 µl of a SYBR green reaction mixture containing 1 µl of cDNA, iQSYBR Green Supermix (Bio-Rad, Hercules, CA), and the respective primers. The following primers were used: ERK5 (Extracellular signal-regulated kinase5) (NM_011840): 5′-ggacaggtcaagctgt gtga-3′, 3′-tggatcccatactgctctcc-5′; PPARγ (Peroxisome proliferator-activated receptor γ) (NM_013124): 5′-catttttcaagggtgccagt-3′, 3′-gaggccagcatggtgtagat-5′; Adiponectin (NM_009605): 5′-gcagagatggcactcctgga-3′, 3′-cccttcagctcctgtcattcc-5′; IL-6 (Interleukin-6) (NM_012589): 5′-gcccttcaggaacagctatg-3′, 3′-gtctcctctccggacttgtg -5′; ap2 (adipocyte lipid-binding protein 2) (AF144756): 5′-atgtgtcatgaaaggcgtga-3′, 3′-aaaccaccaaatcccatcaa-5′; Runx2 (Runt-related transcription factor 2) (XM_001066956): 5′-aagtgcggtgcaaactttct-3′, 3′-aaatgactcggttggtctcg-5′. 18s was used as the housekeeping gene. 18s (M11188): 5′-gcaattattccccatgaacg-3′, 3′-ggcctcactaaaccatccaa-5′.

#### miRNA detection

Levels of miR-143 (regulator of adipogenic pathway) and miR-204 (regulator of osteogenic pathway) were quantified in the total RNA fraction isolated from the preadipocytes (5×10^5^) using miRNA detection kit (AM1558) and respective primers: miR-143 (AM30045) and miR-204 (MIMAT0000877) on the MyiQ Bio-Rad real-time PCR system. MirVana normalization primer set (5s) (AM30302) (Ambion, Austin, TX) was used for normalization.

Results for mRNA and miRNA were calculated using the Pfaffl method (2^-ΔΔCt^) and expressed as fold change of relative expression: mean±SEM of the experimental gene compared to the housekeeping gene in each group [36]. The differences in mRNA or miRNA expression were analyzed by the levels of expression of any particular mRNA or miRNA in 30 mo old group compared to the control 6 mo old group of rats.

### Western blot

Total protein in the cell lysates was quantified using the Lowry method [37]. Approximately, 25 µg cell proteins were subjected to SDS-PAGE gel electrophoresis. After transfer onto nitrocellulose membranes, blots were probed individually with a solution of rabbit polyclonal antibody to rat ERK5 (1∶7000) (#3372, Cell Signaling. Danvers, MA), PPARγ (1∶3000) (600-401-419; Rockland, Gilbertsville, PA), Runx2 (1∶2000) (sc-10758, Santa Cruz Biotechnology, Santa Cruz, CA) or β-actin (1∶1000) (Sigma, St. Louis, MO) as housekeeping protein and then analyzed using the chemiluminescence detection method. The data are expressed after densitometry using Image J as a ratio of the relative density of the protein of interest to housekeeping protein.

### Gain or loss of function analysis of the role of miR-143

To validate the physiological relevance of miR-143 in the regulation of adipogenic pathway during aging, the isolated preadipocytes from young (6 mo) and old (30 mo) rats were transfected with either premir-143 (AM17100) to increase miR-143 levels or with antagomir-143 (AM17000) to decrease miR-143 levels in cells cultured in 6-well plates (50,000 cells per well). Transfection was performed using the lipofectamine-2000 kit (Promega, Madison, WI) following the manufacturer's protocol. After 24 hrs of transfection, the cells were collected in either Tri-reagent (RT-PCR) or RIPA buffer (Western Blot) for further analyses.

### Statistics

For the RT-qPCR analysis all statistics were performed at the level of DCt, in order to exclude potential bias due to averaging of data transformed through the Pfaffl equation 2^-(DDCt)^. The differences of mRNA or miRNA between groups were determined by one-way ANOVA using the SPSS statistical package (Chicago, IL, USA). A probability value of <0.05 was considered statistically significant [38]. The differences in protein levels (as assessed by protein band density) were analyzed by one-way ANOVA using SPSS, compared to the control. The p value of <0.05 was defined as significant. Significance was confirmed using post-hoc analysis using Fisher's least significant difference (Fisher's LSD) test.

## Results

### SVF cells characterization

**Figure 1:**
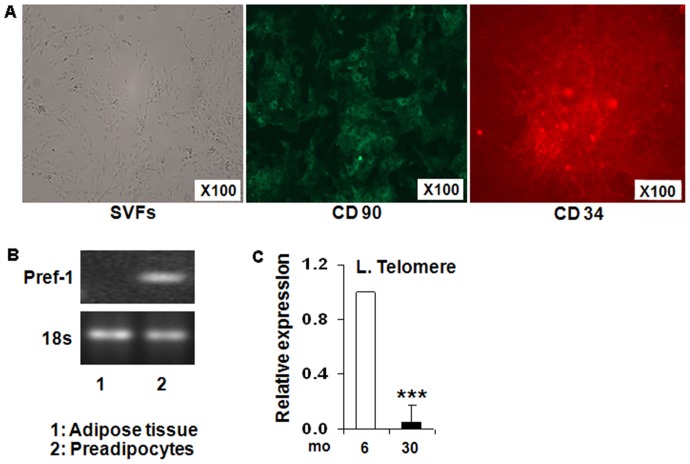
Morphological characterization of preadipocytes isolated from the abdominal visceral fat from young (6 mo) and old (30 mo) FBN Rats. Stromal vascular fraction *(SVF)* was isolated and fractionated from the visceral adipose tissue obtained from both groups (n = 8/group) of rats. Preadipocyte fraction (ASCs) was characterized by immunostaining of cell surface markers, CD90 and CD34 in SVF cultured cells, ***1A***; Higher mRNA levels of Pref-1 (preadipocyte marker) in SVF cells and not in whole abdominal fat tissue as measured by RT-qPCR, ***1B***; Increased telomere length (senescent marker) in SVF cells isolated from 30 mo rats and not in 6 mo rats, ***1C***.

The SVF fractions isolated from the adipose tissue were cultured in 6-well cell culture dishes for over a week. The cells that survived 3–5 passages of the SVF cells were mostly fibroblast-like (preadipocytes). The morphology of SVF (stromal vascular fraction that contain preadipocytes-ASC) isolated from both young (6 mo) and old (30 mo) rats were confirmed by a positive immune-fluorescent staining of CD90, but a minimal staining of CD34 **(** **Figure 1A** **)**. Similarly in **Figure 1B**, Pref-1 (preadipocyte marker) was detectable only in the SVF containing preadipocytes but not in the whole adipose tissue.

**Figure 1C** showed that preadipocytes isolated from older rats expressed a significantly shorter telomere length (a marker for senescence) compared to the preadipocytes isolated from younger rats (-90%, p<0.005), suggesting the senescent nature of the preadipocytes isolated from the aged rats.

As an additional method of characterizing the stem cell markers in the isolated SVF derived cells, flow cytometry was used to detect CD90, CD34 and CD44 positive cells. FlowJo 10.1 was used for the quantitation of the CD cell surface markers. As shown in the Supplementary **Figure S1A**, there were equal numbers of CD positive cells in the two rats (81.6% vs 90.7% cells in 6 mo vs. 30 mo rats). Among the three markers that were stained, there was CD34+(51.3% vs 90.5%), CD90+(90.7% vs 51.3%) and CD44+(51.4% vs 90.8%) staining in young (6 mo) versus old (30 mo) SVF derived cells. There seems to be a switch from CD90+cells to CD34+cells with aging. Supplementary **Figure S1B and C**, also showed 51% of either CD90+/CD34+ or CD44+/CD34+double positive cells in young (6 mo) rats compared to 90% of CD90+/CD34+and CD44+/CD34+ double positive cells in SVF derived cells from old (30 mo) rats.

### Aging altered adipogenic and osteogenic differentiation capacity of preadipocytes

**Figure 2:**
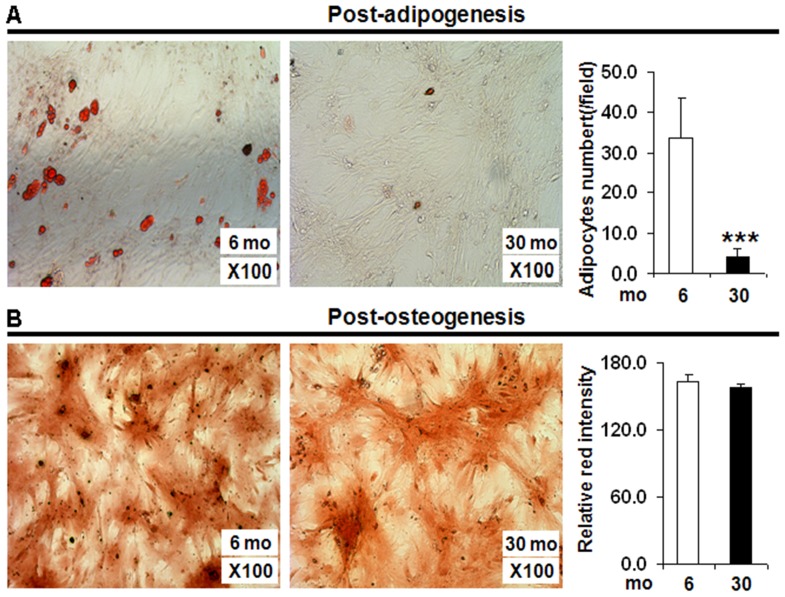
Age associated changes in adipogenic or osteogenic differentiation capacity of SVF cells. The SVF derived preadipocytes isolated from 6 mo old (n = 8) and 30 mo old (n = 8) FBN rats were subjected to either adipogenic or osteogenic differentiation protocol. A representative figure demonstrating the increased adipogenic differentiation potential of preadipocytes from 6 mo rats compared to older (30 mo) rats as exhibited by the presence of fat red (oil droplets of adipocytes) staining, ***2A***; A representative figure demonstrating equal osteogenic differentiation potential of preadipocytes isolated from both groups of rats, as seen by positive alizarin (mineralization) staining for osteogenesis, ***2B***. The number of fat red positive and alizarin positive cells were quantified using IMAGE-J software. The preadipocytes from 6 mo rats were defined as control (CTRL). *: p<0.05.

The SVF cells isolated from both young (6 mo) and old (30 mo) rats were subjected to adipogenic or osteogenic differentiation in culture. As shown in Figure 2A, the SVF cells from old rats (30 mo) displayed a significant reduction in its ability to undergo adipogenic differentiation as observed by lower number of fat red stained mature adipocytes compared to the adipocytes derived from the differentiation of preadipocytes from young (6 mo) rats. In contrast, in Figure 2B SVF cells from both rats exhibited an equal osteogenic differentiation capacity as displayed by similar mineralization (alizarin positive cells) staining between the two groups.

### Altered expression of miRNAs that regulate adipocyte or osteocyte differentiation with aging

**Figure 3:**
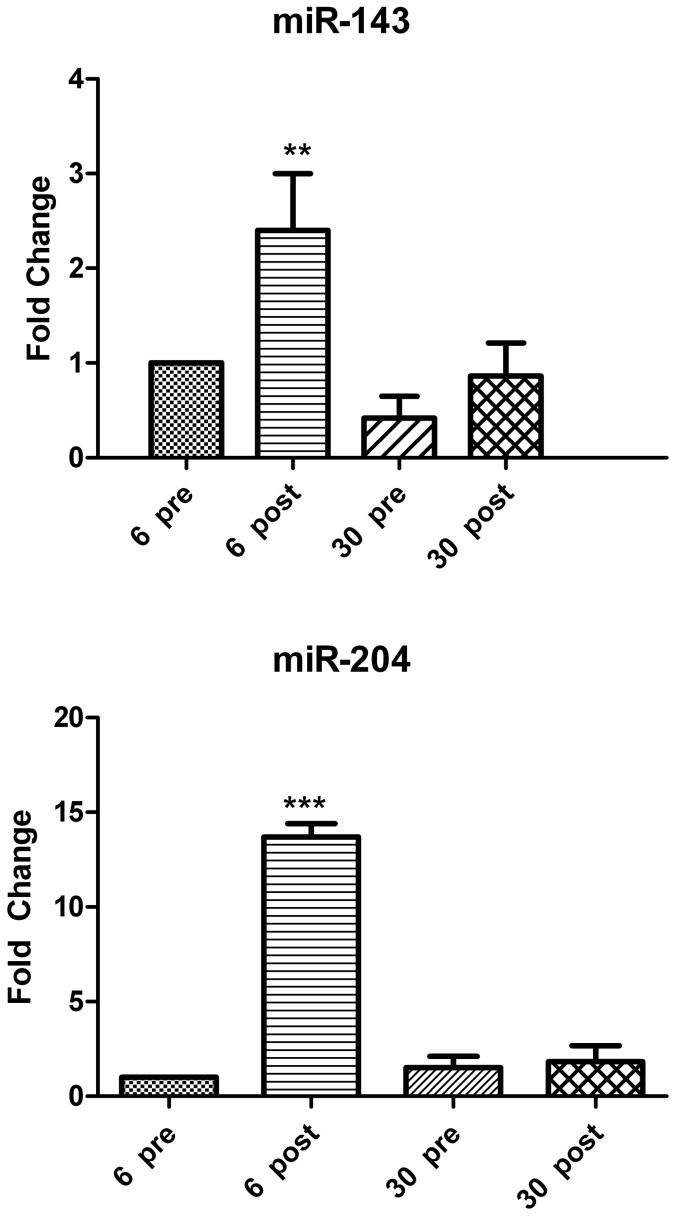
Adipogenic and osteogenic miRNA expression in SVF cells. MiR-143 that regulates adipogenic differentiation and miR-204 that regulates osteogenic differentiation were measured in SVF cells isolated from 6 mo and 30 FBN rats (n = 8/group) before and after adipocyte differentiation using mirVana assays. An increase in miR-143 and miR-204 after adipocyte differentiation in 6 mo rats but not in 30 mo rats was observed. miR-143, ***3A***; miR-204, ***3B***. The RT-qPCR data was expressed as fold of relative expression±SEM (Standard Error of Mean). Preadipocytes from 6 mo rats were defined as control. **: p<0.01; ***: p<0.005.

To investigate if miRNAs that regulate adipocyte or osteocyte differentiation is compromised in SVF cells from old rats (30 mo), the levels of miR-143 [26] and its target gene ERK5 and miR-204 (which directly inhibits osteogenic factor-Runx2) [27] were determined in the preadipocytes isolated from both groups of rats before (baseline) and after subjecting to *ex-vivo* adipocyte differentiation. As observed in **Figure 3A and B** **,** the levels of both miR-143 and miR-204 were not significantly different in the young and old preadipocytes before adipocyte differentiation (pre-adipogenesis). However, after differentiation, only the young cells (6 mo rats) had significantly higher expression of miR-143 (2.4 fold, p<0.01) and miR-204 (13.7 fold, p<0.005) (post-adipogenesis) (**Figures 3** **&** **4** **A and B**). The adipogenic differentiation did not alter the miRNA levels in old SVF cells (30 mo rats).

**Figure 4:**
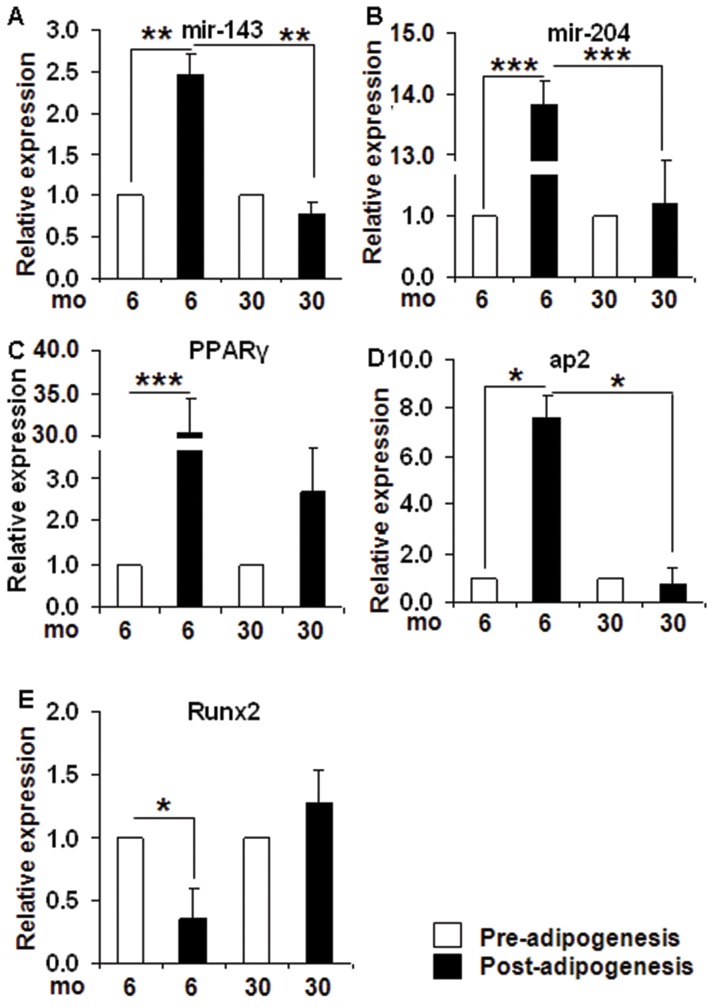
Age associated changes in adipogenic or osteogenic miRNAs and their target genes in SVF cells before and after adipogenic differentiation. MiR-143 that regulates adipogenic differentiation and miR-204 that regulates osteogenic differentiation and their target genes, were measured in SVF cells isolated from 6 mo and 30 rats (n = 8/group) before and after adipocyte differentiation using mirVana assays. miR-143, ***4A***; miR-204, ***4B***; PPARg, ***4C***; ap2, ***4D***; and Runx2, ***4E***. The RT-qPCR data was expressed as fold of relative expression±SEM (Standard Error of Mean). Preadipocytes from 6 mo rats were defined as control. *: p<0.05; **: p<0.01; ***: p<0.005.

**Figure 5:**
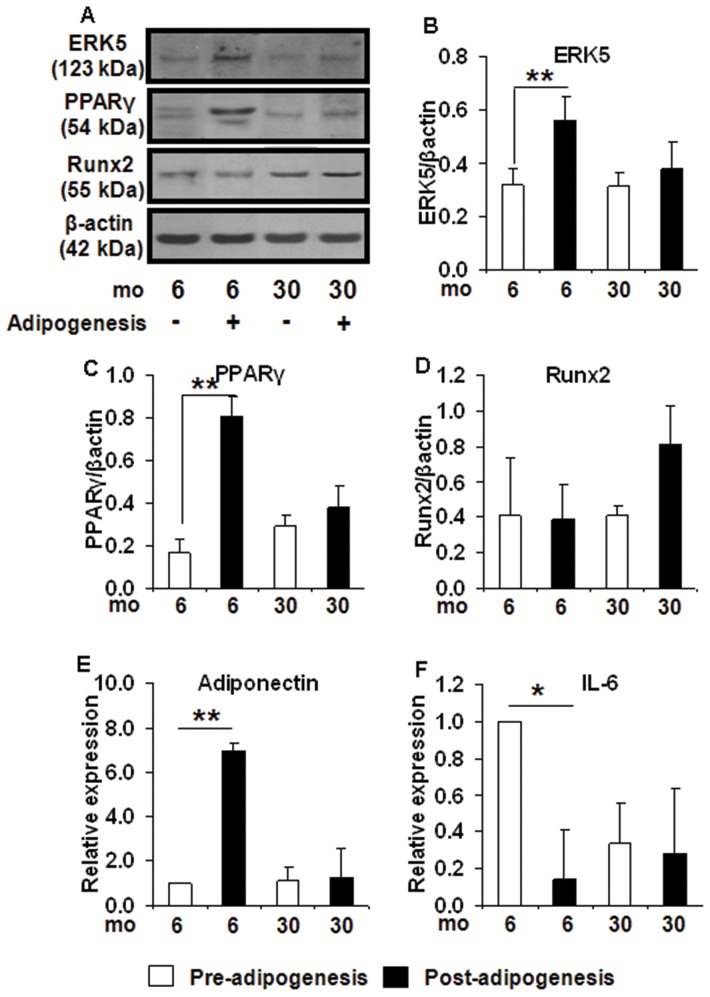
Impaired protein expression of miR-143 target genes with aging in preadipocytes before and after adipogenic differentiation. A decreased adipogenic differentiation potential of preadipocytes from old rats (30 mo) compared to 6 mo (n = 8/group) rats was due to changes in miR-143 levels. Western blot was used to analyze the protein levels of miR-143 target genes, ERK5, *5A* and *B*; PPARg, *5A* and *C*; or osteogenic marker, Runx2, *5A and D* in the young and old rats preadipocytes before and after adipogenic differentiation. RT-qPCR was also used to measure adipogenic markers, adiponectin, *5E* and IL-6, *5F*. The data was expressed as ratio or fold of relative expression±SEM (Standard Error of Mean). *: p<0.05; **: p<0.01.

Correspondingly, a significant induction in the protein levels of the target gene for miR-143, ERK5 (+78%, p<0.01) (**Figure** **5A and 5B**), which in turn regulates adipogenesis by modulating PPARγ [39] [26] mRNA (30.6 fold, p<0.005) (**Figure** **4C**) and protein (+370%, p<0.01) (**Figure** **5A and 5C**) was observed in preadipocytes from young (6 mo) rats post-adipogenesis compared to old (30 mo) rats. Compared to the preadipocytes from the younger rats, the preadipocytes isolated from 30 mo rats expressed much lower PPARγ and ap2 (adipogenic marker) (**Figure** **4C and 4D**) post-adipogenesis. In contrast, a reduction in Runx2 (osteogenic marker) mRNA (0.36 fold, p<0.05) (**Figure** **4E**) but not its protein (**Figure 5D**) in 6 mo cells post-adipogenesis, but not in 30 mo preadipocytes was observed.

### Altered expression of adipose derived factors in preadipocytes from older rats

**Figure 6:**
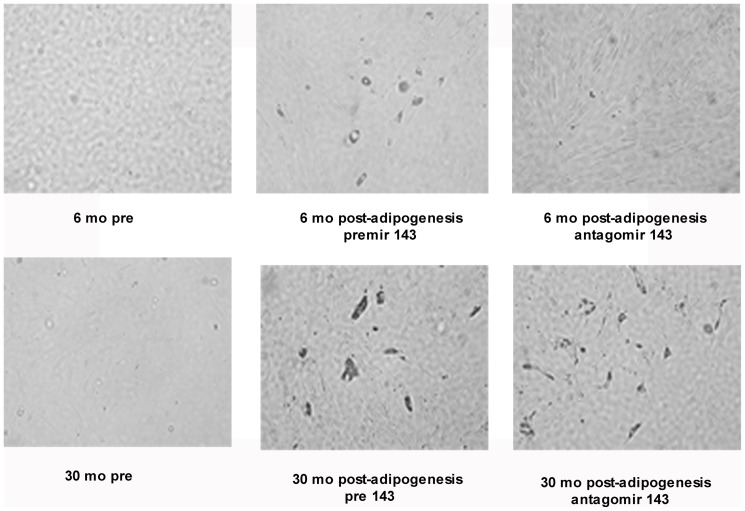
Morphological characterization of SVF cells isolated from the abdominal visceral fat from young (6 mo) and old (30 mo) FBN rats after miR-143 premir and antagomir transfection. SVF cells from both young (6 mo) and old (30 mo) rats were transfected with premir-143 or antagomir-143. Cell morphology was analyzed using microscopy before and after adipogenic induction. Presence of lipid droplet containing cells (mature adipocytes) in 6 mo cells post induction after premir-143 transfection but not after antagomir-143 transfection was observed. No such mature adipocytes were observed in 30 mo SVF cells with either transfection.

In order to investigate if changes in miR-143 levels with age also compromised adipose function, the synthesis and secretion of adipose derived factors, adiponectin and IL-6 were determined. There was an induction of adiponectin (anti-inflammatory) mRNA expression (6.96 fold, p<0.01), but a down-regulation in IL-6 (pro-inflammatory) expression (0.14 fold, p<0.05) in 6 mo preadipocytes post-adipogenesis (**Figure 6E and 6F**) however, no such changes were observed even after adipogenesis in preadipocytes from 30 mo old rats.

### Validation of the role of miR-143 in age-impaired adipogenesis

Results thus far indicated that in contrast to preadipocytes from young rats, preadipocytes isolated from visceral fat from older rats (30 mo) have a lower capacity to undergo adipocyte differentiation and a lower ability to synthesize and secrete adipose derived factors even after adipocyte induction. This dysfunction was associated with compromised miR-143 (known to play a regulatory role in adipocyte differentiation) levels. The regulatory role of miR-143 in the adipogenic/osteogenic pathways with age was validated by modulating the miR-143 expression levels in preadipocytes from both young (6 mo) rats and old (30 mo) rats after transfection with either premir (gain of function) or antagomir-143 (loss of function) primers. After transfection, cells were differentiated into adipocytes using adipocyte specific differentiation medium (post-adipogenesis). Though, the morphology of the cells from both groups of rats was intact before and after transfection and adipogenic induction (**Figure 6**), their ability to differentiate into mature adipocytes were altered with aging. There was presence of lipid filled cells (marker of adipocytes) in young cells after premir-143 transfection, but no such cells were seen after antagomir-143 transfection. Though cell morphology did not alter after transfection in 30 mo SVF cells, no such differentiation into mature adipocytes (lipid filled cells) was observed post adipogenic induction in these cells.

**Figure 7:**
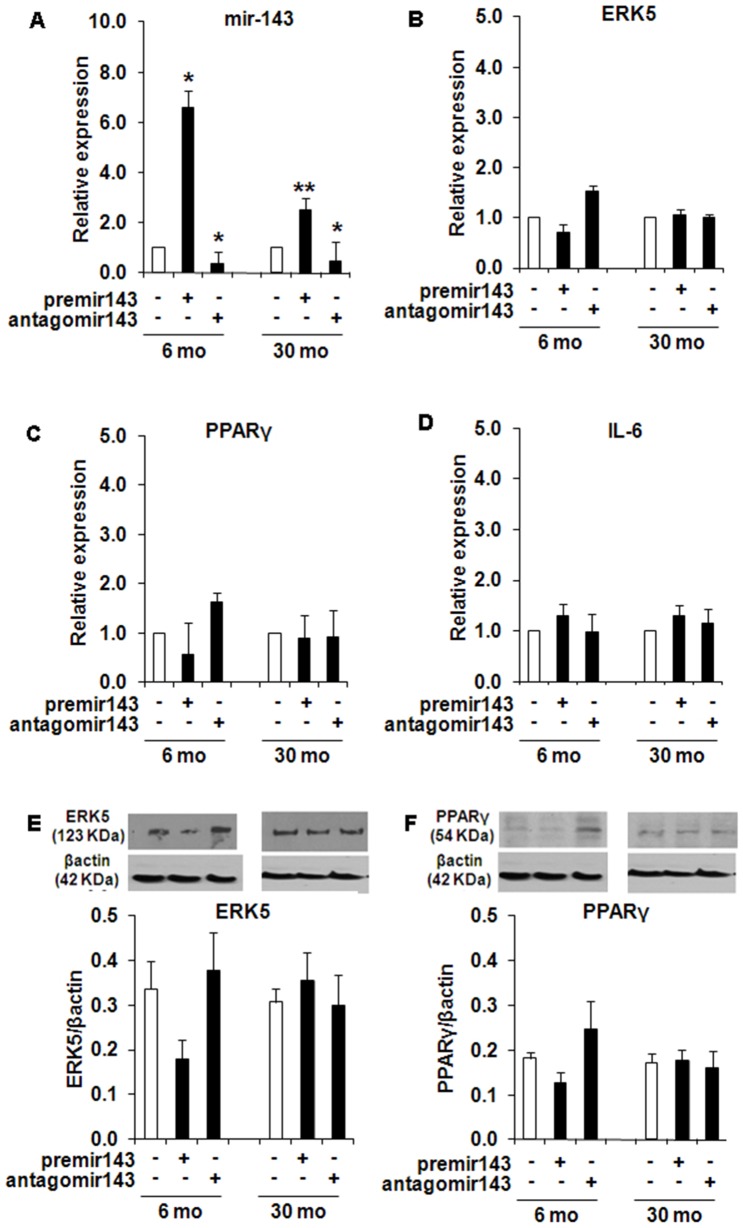
Gain and loss of function assays to validate the role of miR-143 in young (6 mo) and old (30 mo) rat SVF cells, pre-adipogenesis. The gain or knockdown of miR-143 by premir or antagomir-143 was performed by transient transfection of both young and old rat (n = 8) preadipocytes, pre-adipogenesis. The transfection efficiency and target gene levels were confirmed by RT-qPCR and Western blot analysis. miR-143, *7A*; ERK5 mRNA, *7B*; PPARg mRNA, *7C*; IL-6 mRNA, 7D; ERK5 protein, *7E*; and PPARg protein, *7F.* The real time PCR data was expressed as fold of relative expression±SEM (Standard Error of Mean). *: p<0.05; **: p<0.01.

Transfection efficiency (**Figure 7A**) was confirmed by a significant induction of miR-143 after transfection with premir-143 in both 6 mo(>6 fold, p<0.05) and 30 mo rat SVF cells (>3.5 fold, p<0.005) and significant decrease in miR-143 levels after antagomir-143 transfection in 6 mo and 30 mo rat(<0.4 fold, p<0.05) SVF cells. Though a decrease in mRNA and protein levels of ERK5 and PPARg (**Figure 7** **B, C, E and F**) were observed after premir-143 transfection in young SVF cells no significant changes were observed in either of the groups after antagomir-143 transfection. No change in IL-6 levels was seen in either group of cells (**Figure 7D**).

**Figure 8:**
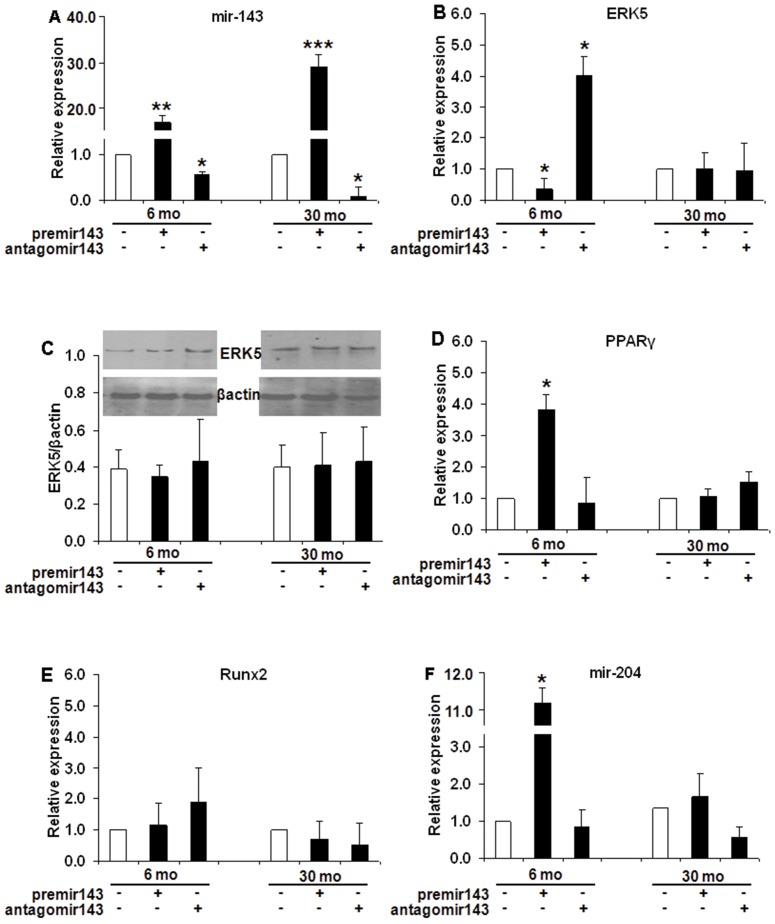
Gain and loss of function assays to validate the role of miR-143 in young (6 mo) and old (30 mo) rat SVF derived cells, post-adipogenesis. Adipocyte differentiation was performed on the cells after transient transfection of both young and old rat (n  = 8) preadipocytes by premir or antagomir-143 (gain or knockdown of miR-143). At the end of 14 days post-adipogenesis, the transfection efficiency and target gene levels were analyzed by RT-qPCR and Western blot analysis. miR-143, *8A*; ERK5 mRNA, *8B*; ERK5 protein, *8C*; PPARg mRNA, *8D*; Runx2, *8E;* and miR-204, *8F.* The real time PCR data was expressed as fold of relative expression±SEM (Standard Error of Mean). *: p<0.05; **: p<0.01; ***: p<0.005.

In transfected SVF derived cells after differentiation into mature adipocytes using adipocyte induction media (post-adipogenesis), the transfection efficiency remained high after transfection with premir-143 in both 6 mo (17.5 fold, p<0.005) and 30 mo rat cells (29.6 fold, p<0.005) and significant decrease in miR-143 levels after antagomir-143 transfection in 6 mo (0.52 fold, p<0.05) and 30 mo rat (0.13 fold, p<0.05) SVF derived cells (**Figure 8A**). Since miR-143 negatively regulates ERK5 mRNA [26], a higher miR-143 levels after premir-143 transfection significantly decreased ERK5 mRNA level (0.36 fold, p<0.05) but increased its levels (4.02 fold, p<0.05) after antagomir-143 transfection (**Figure 8B** **)** post-adipogenesis, only in 6 mo SVF derived cells but not in 30 mo rat cells. Similarly, there was also a significant induction in PPARg mRNA levels (3.75 fold, p<0.05) after premir-143 transfection in SVF derived cells post-adipogenesis from younger but not older rats but no change after antagomir-143 in both cells, **Figure 8D**. However, no significant changes in ERK5 protein levels were observed in either age group after adipogenesis, **Figure 8C**. The lower levels of Runx2 mRNA levels with premir-143 in young rat SVF derived cells (**Figure 8E**) was probably due to the significant induction of miR-204 (11.18 fold, p<0.05) (**Figure 8F**) in these cells post-adipogenesis. However, no such changes were observed post-adipogenesis with antagomir-143 transfection in SVF derived cells from young rats or by either manipulations in cells from aged rats.

## Discussion

Adiposity increases as humans' age. Similar phenotypic changes are also observed in FBN hybrid rats as they become old (>26 mo) [40]. This increase in age-related fat accumulation in humans [41] and rodents is mainly attributed to altered insulin sensitivity, which is a major risk factor for obesity, diabetes and atherosclerosis. Understanding the role of RNA regulatory mechanisms influenced by aging and its impact on age-related diseases is an area of intense investigation. Despite growing evidence in the literature on the role of miRNAs on RNA regulatory mechanisms, its potential role in age-related decline in function has not been well investigated. Alterations in miRNAs have been shown in rodents and humans with senescence or increasing age. MiRNAs such as hsa-let-7f, hsa-miR-499, hsa-miR-373, hsa-miR-372, hsa-miR-371, hsa-miR-369-5p, hsa-miR-34c, hsa-miR-34b, hsa-miR-34a, hsa-miR-29c, hsa-miR-217, and hsa-miR-20a might influence senescence or aging [42]. Similarly, miRNAs that influence adipogenesis or osteogenesis pathways have been studied [23], [24]. However, no studies have correlated miRNA regulation on adipose tissue function with age.

The aging process independently influences adipose tissue morphology, distribution and function [2]. The higher ratio of central versus peripheral fat is attributed to weight gain [43] with aging. We recently showed that the gene expression of various adipose derived factors was dramatically altered with age and was dependent on both the source of the fat depot (visceral versus epicardial fat) and gender [18]. These fat depot changes observed with age are highly correlated to the dysfunction of the SVF derived stem cells [17]. The gene regulation and secretion profiles of SVF derived stem cells also dramatically influence fat tissue function [17].

The ability to undergo adipogenic, chondrogenic and osteogenic differentiations constitute the elementary capacity of multi-lineage potential of mesenchymal stem cells, including adipose SVF derived stem cells [5]–[7]. The reciprocal differentiation pathway to adipocyte or osteocyte is switched through activation of either PPARg [44] or Runx2 [21]. These regulators exist in the mesenchymal stem cells and other progenitor cells [45]. Moreover, these two differentiation pathways are found to reciprocally inhibit each other [46]–[48]. Studies have shown that preadipocytes retain their aging phenotype in culture, but are altered in their capacity to undergo adipogenesis and osteogenesis [28] [3]. The senescent preadipocytes with lower adipogenic capacity express decreasing levels of C/EBPa, C/EBPd, and PPARg expression [17]. Any alterations in preadipocyte gene regulation result in fat redistribution and dysfunction during aging [49]. Perturbation in lipid metabolism in preadipocytes enhances lipotoxicity and impairs adipogenesis and lipid oxidation with age [50]. These inherent differences in the nature of adipose SVF derived stem cells determine the differences in fat depot function due to overfeeding [51]. Regulators involved in phenotypic or functional changes in preadipocytes are not known. Since miRNAs regulate adipogenic and osteogenic pathways, our findings support their role in the aging mediated switch in differentiation capacities of preadipocytes.

Reciprocal induction of miR-143 and its target gene ERK5 plays an important role in adipocyte differentiation [26]. The activation of ERK5 enhances and activates PPARg through the interaction of the hinge-helix 1 region of PPARg and ERK5 [39]. In our study, there was a down-regulation of miR-143 levels post adipogenesis, in adipose SVF derived cells isolated from older rats compared to those from younger rats. In addition, the gain or knockdown of miR-143 only altered ERK5 mRNA levels in adipose SVF derived cells from younger rats, but not from 30 mo old rats, with very little change in ERK5 protein expression in adipose SVF derived cells from both groups of rats, suggesting a dysregulation of miR-143 with age. ERK5 is responsible for insulin induced adipogenesis [52]. The observed increase in ERK5, PPARg, ap2 and adiponectin levels in 6 mo old rat adipose SVF derived cells post-adipogenesis, but not in older rats supports the active role of ERK5 during adipogenesis in young cells. However, although miR-143 inhibited ERK5, the levels of the adipogenic factors were increased after adipogenesis in adipose SVF derived cells from young rats. This may possibly be due to a negative feedback regulation by the adipogenic factors to maintain differentiation. Increases in miR-143 levels by premir transfection enhanced the adipogenic differentiation capacity of young adipose SVF derived cells with a concomitant increase in PPARg and miR-204.

Our data also showed that adipose SVF derived cells from 30 mo rats expressed significantly higher Runx2 mRNA level, which may indicate the phenotypic switch during aging in adipose SVF derived cells. Moreover, miR-204 was activated post-adipogenesis in 6 mo SVF derived cells to inhibit Runx2; however, this process was not seen in older rats, resulting in increased levels of Runx2 in these cells. Similarly, a failure of PPARg induction was also observed in the 30 mo cells after adipogenic induction. This supports the hypothesis that miRNA mediated adipogenic differentiation was impaired in aging preadipocytes which was accompanied by an inability of adipogenic miR-204 to suppress RunX2 and other factors (PPARg).

Interestingly, it seems like aging utilizes opposing mechanisms to influence progenitor cell function in adipose tissue and bone marrow. Contrary to what was observed in this study, the mesenchymal stem cells in bone marrow were found to exhibit activated adipogenic but suppressed osteogenic capacity, thus contributing to osteoporosis in seniors [53]. In addition, it is possible that the differences in CD markers (switch in number of CD90+versus CD34+cells) in young versus old SVF derived cells might play a role in the phenotypic switch observed in our studies with aging.

Our findings suggest a new regulatory mechanism in adipose function during the aging process. An impairment of adipogenic program accompanied with an unregulated osteogenic program, influences preadipocyte perturbation in the aging process. This phenomenon correlated with the impairment of the miRNA regulatory pathway. Moreover, unresponsiveness to miRNA intervention by the adipose SVF derived cells from old rats (30 mo) suggested that miRNA dysregulation contributed to the pathological processes observed in certain tissues during aging. This dysregulation might be attributed to epigenetic alterations in both miRNA and its target messenger RNA (mRNA), alterations in the proteins involved in miRNA biogenesis or due to alternate regulatory phenomenon such as miRNA editing.

## Supporting Information

Figure S1 **Analysis of adipose SVF derived cells by flow cytometry.** SVF derived cells from 6 mo and 30 mo FBN rats were incubated with antibodies to cell surface markers CD90, CD34 and CD44 and analyzed using BD FACS Aria Flow Cytometry followed by quantitation using FlowJo 10.0 software. ***1A***. Density plot and histogram of population of cells that stained positive for all markers (Total), or individually for CD34, CD90 or CD44 in SVF derived cells from 6 mo and 30 mo FBN rats; ***1B***. Density plot displaying the percentage of dual positive markers for PCP-Cy5- conjugated CD34 and PE-conjugated CD90 (***1*** **B)** or PCP-Cy5- conjugated CD34 and FITC-conjugated CD44 (***1C***) in SVF derived cells from either 6 mo (***1B and C-i***) or 30 mo (***1B and C-ii***) FBN rats. The total number of positive or negative stained cells is given as percentages.
(TIF)
